# *Rickettsia parkeri* Infection in Domestic Dogs, Southern Louisiana, USA, 2011

**DOI:** 10.3201/eid1806.120165

**Published:** 2012-06

**Authors:** Britton J. Grasperge, Wendy Wolfson, Kevin R. Macaluso

**Affiliations:** Louisiana State University, Baton Rouge, Louisiana, USA

**Keywords:** Rickettsia parkeri, canine, dog, Louisiana, tick-borne rickettsial disease, bacteria, Rickettsia, arthropod vectors, companion animals, rickettsioses, Rocky Mountain spotted fever, spotted fever group rickettsioses

## Abstract

The association between companion animals and tick-borne rickettsial disease has long been recognized and can be essential to the emergence of rickettsioses. We tested whole blood from dogs in temporary shelters by using PCR for rickettsial infections. Of 93 dogs, 12 (13%) were positive for *Rickettsia parkeri*, an emerging tick-borne rickettsiosis.

Tick-borne spotted fever group (SFG) rickettsioses are maintained in tick populations through vertical transmission of the rickettsial agent and horizontal transmission among vectors by a vertebrate host. Companion animals, specifically dogs, can serve as vertebrate hosts for arthropod vectors and SFG rickettsia (*1*), as shown by a report of a *Rickettsia parkeri*–infected dog in South America (*2*). Likewise, cases of rickettsioses in humans have been associated with cases in companion animals (*3*). Because of a substantial increase in tick-borne rickettsial diseases in the past decade, much effort has been directed to identifying the rickettsial agents present in ticks (*4*). On the basis of findings from field surveys of rickettsial infections in ticks and characterization of rickettsioses in humans, most cases of what is considered Rocky Mountain spotted fever, a disease caused by *R. rickettsii*, are likely caused by infections with rickettsial species other than *R. rickettsii* (*5*).

One of the better documented emerging rickettsial pathogens is *R. parkeri*, an SFG tick-borne rickettsial disease associated with Gulf Coast ticks (*Amblyomma* *maculatum*) (*6*) and commonly identified in the coastal states of the southeastern United States. We investigated the potential role domestic dogs play in the ecology of *R. parkeri* transmission to better understand the epidemiologic landscape of this emerging rickettsiosis.

## The Study

We obtained blood from dogs at 5 animal control centers in 5 parishes in southern Louisiana during June and July 2011. The blood for the study was provided from excess samples collected for routine heartworm screening. In total, 93 dogs were included in the study. Within 12 hours of collection, whole blood samples (≈50–100 μL) were processed individually for DNA extraction by using the DNeasy Blood and Tissue Kit (QIAGEN, Valencia, CA, USA). DNA was stored at –20°C until PCR analysis.

DNA extracts from the collected blood, environmental DNA extraction controls, or water (negative controls) were used as template for PCR. PCR products were amplified by using genus-specific 17-kDa antigen gene primers and described thermocycling conditions (*7*). Amplicons were visualized by electrophoresis on 2% agarose gels. Positive samples were excised from the gels, and the amplicons were purified by using the PCR Clean-Up System (Promega, Madison, WI, USA). Positive samples were sequenced, and sequences were aligned by using MEGA5.05 (http://megasoftware.net/mega.php), and nucleotide similarities were assessed by using the GenBank BLAST database (http://blast.ncbi.nlm.nih.gov/Blast.cgi).

DNA samples positive for *Rickettsia* spp*.* by the genus-specific 17-kDa antigen gene primers were also assessed for the SFG-common rickettsial outer membrane protein A gene (*rompA*) by using a heminested PCR with primers 190.70p and 190.701 followed by primers 190.70p and 190.602n. Primers and thermocycling conditions for the heminested PCR were as described (*7*), and subsequent purification and sequencing were performed as described above.

Of the 93 DNA samples, 12 (≈13%) produced positive amplicons for the genus-specific 17-kDa antigen gene. On the basis of sequence data, the positive samples were determined to be most closely related to SFG rickettsiae. The resulting 315-bp sequence showed 100% identity to *R. montanensis* (GenBank accession no. DQ402377.1) and 99% identity to several other members of the SFG including *R. rickettsii*, *R. parkeri*, *Candidatus* Rickettsia andeanae, and *R. sibirica* (GenBank accession nos. CP000766.2, EF689732.1, GU395295.1, and AF445384.1, respectively).

The heminested PCR for *rompA* yielded a 491-bp product with identical sequences for each of the 12 *Rickettsia*-positive samples. Sequence analysis of the *rompA* amplicon identified a 99% similarity with several different strains of *R. parkeri* (GenBank accession nos. U43802.1, EU715288.1, EF102238.1, FJ172358.1, and HM587252.1). These *Rickettsia*-positive samples were obtained from 3 of the 5 sites surveyed, and 2 of the 3 sites were in parishes that directly adjoined each other (Table). Within the dog populations tested in the 3 sites, 22% (2/9), 16% (9/55), and 8% (1/12), respectively, of the dogs were infected with *R. parkeri* (Table).

**Table:** *Rickettsia parkeri* infection in domestic dogs in 5 animal shelters from 5 parishes in southern Louisiana, USA, June and July 2011*

Parish	No. dogs positive/no. tested (%)
Ascension	0/13
Livingston†	2/9 (22)
Iberville	0/4
East Feliciana	9/55 (16)
Tangipahoa†	1/12 (8)

*The presence of *R. parkeri* was determined by PCR for *rompA.* *R. parkeri*, *Rickettsia parkeri*; rompA, rickettsial outer membrane protein A. †Parishes directly adjoin one another.

None of the 12 dogs with PCR-positive tests were infested with ticks at the time of sampling. Six female dogs and 6 male dogs had detectable levels of *R. parkeri* DNA in their blood. Nine of the 12 dogs were adults; 3 were <6 months of age. Many of the dogs in the study were classified as mixed breed because breed could not be objectively determined for most of the animals. All animals appeared to be in good health; no overt pathology was noted at the time of blood collection.

Although molecular detection of rickettsial DNA within the blood of vertebrates indicates infection, rickettsial cultures from the positive samples would confirm patent rickettsemia. Most of the samples in our study were insufficient in volume to attempt culture after heartworm testing and DNA extraction. Of the 12 samples with PCR results positive for rickettsial DNA, only 3 were of sufficient volume to attempt culture, and all of those attempts proved unsuccessful. It would also have been beneficial to determine if dogs that were positive for *R. parkeri* harbored ticks that were also positive for *R. parkeri*. However, it is common practice for animal control centers to treat dogs for ectoparasites on admission; thus, no ticks were present on the dogs in our study at the time of sampling. The presence of rickettsial DNA in the blood of dogs, in the absence of ectoparasites, supports the hypothesis that domestic canines may serve as reservoirs of rickettsial diseases, now specifically including the emerging pathogen *R. parkeri*.

## Conclusions

We examined the potential role of domestic dogs in transmission of SFG *Rickettsia*. *R. rickettsii*, the causative agent of Rocky Mountain spotted fever, is known to cause clinical disease in dogs, and it is associated with signs and symptoms that are similar to human disease, including cutaneous petechiae and ecchymoses, anorexia, depression, weight loss, and dehydration (*8*). The role of dogs as vehicles for *Rickettsia*-infected ticks to encounter susceptible humans has also been proposed (*1*). The possibility of dogs as reservoirs of rickettsial disease has previously been investigated in studies evaluating *R. felis* rickettsemia and seropositivity for *R. parkeri* (*9**,**10*); however, strong cross-reaction among antibodies precludes finding of definitive results from serologic testing. The current study suggests that domestic dogs may become rickettsemic with *R. parkeri* infection, but further investigation of the duration of rickettsemia and monitoring for clinical disease that may be associated with infection is required.

It is also vital to determine the potential for dogs to serve as infectious sources of *R. parkeri* for feeding ticks. Dogs infected with *R. rickettsii*, for example, have proven relatively inefficient at transmitting rickettsiae to naive ticks and therefore may not play a large role in maintenance or amplification of the *R. rickettsii* transmission cycle (*11*). Conversely, domestic dogs have recently been shown to be competent reservoirs for the causative agent of Mediterranean spotted fever, *R. conorii*, a species closely related to *R. parkeri* (*12*). The prevalence identified in this study establishes an important first step in the examination of the domestic dog for reservoir competency of *R. parkeri*.

Since the first reported case of *R. parkeri* rickettsiosis in 2004, >20 additional cases have been identified in humans (*13*), and to date no viable vertebrate reservoirs for the pathogen have been identified. Although the current study consists of a relatively small survey, the results are considerable because of the recognized importance of domestic dogs as potential reservoirs for transmissible pathogens (*14*). In addition, the presence of *R. parkeri* has not previously been described in Louisiana; thus, this report expands the known distribution of *R. parkeri*. The results of the current study clearly establish dog infection by *R. parkeri*; however, a role for dogs in the natural cycle of this pathogen, and the arthropod vectors involved in transmission, requires further investigation.
